# Design of a Measuring System for Electricity Quality Monitoring within the SMART Street Lighting Test Polygon: Pilot Study on Adaptive Current Control Strategy for Three-Phase Shunt Active Power Filters

**DOI:** 10.3390/s20061718

**Published:** 2020-03-19

**Authors:** Radek Martinek, Petr Bilik, Jan Baros, Jindrich Brablik, Radana Kahankova, Rene Jaros, Lukas Danys, Jaroslav Rzidky, He Wen

**Affiliations:** 1 Department of Cybernetics and Biomedical Engineering, Faculty of Electrical Engineering and Computer Science, VSB–Technical University of Ostrava, 17. listopadu 15, 708 33 Ostrava, Czech Republic; rade.martinek@vsb.cz (R.M.); petr.bilik@vsb.cz (P.B.); jan.baros@vsb.cz (J.B.); jindrich.brablik@vsb.cz (J.B.); radana.kahankova@vsb.cz (R.K.); lukas.dany@vsb.cz (L.D.); jaroslav.rzidky@vsb.cz (J.R.); 2 College of Electrical and Information Engineering, Hunan University, Lushan Road, Yuelu District, Changsha 410082, China; he_wen82@126.com

**Keywords:** power quality (PQ), shunt active power filter (SAPF), virtual instrumentation (VI), BROADBAND^LIGHT^, least mean squares filter (LMS), recursive least squares filter (RLS)

## Abstract

This study focuses on the design of a measuring system for monitoring the power quality within the SMART street lighting test polygon at university campuses with relation to testing an adaptive current control strategy for three-phase shunt active power filters. Unlike conventional street lighting, SMART elements are powered 24/7. Due to the electronic character of the power part of such mass appliances, there are increased problems with the power quality of the electric energy. Compared to the current concept of street lighting, there is a significant increase in the content of higher current harmonic components, which cause several problems in the distribution system. The test polygon contains 16 luminaires made by various manufacturers and mounted with various SMART components. Using the polygon control and monitoring system, dynamic load scenarios were selected. These scenarios tested the possibilities of different adaptive current control strategies for three-phase shunt active power filters to improve the power quality of electricity. This study focuses on three adaptive algorithms that respond to dynamic changes of current harmonics level in real-time. The possibility of active filter control was tested using FPGA, mainly due to the low latency of the filter control part.

## 1. Introduction

Street lighting is responsible for up to 15% of the national energy consumption [1]. From the perspective of current technologies, the operation of street lighting luminaires is usually very inefficient. Conventional light sources hitherto used for street lighting often operate without the possibility of regulation and illuminate at maximum intensity even when it is not necessary [2,3]. They often emit light even when natural ambient light is fully sufficient for the operation of the infrastructure. This leads to unnecessarily higher operating costs [1,4]. There are several methods of reducing the losses. The most radical method of reducing losses is the general replacement of conventional lighting technology with modern technology [5]. In the case of the test polygon in the VSB–Technical University of Ostrava campus described here, it is a transition from sodium vapor lamps to LED lamps. It is predicted that, between 2020 and 2025, the existing conventional street lighting (predominantly sodium vapor lamps) will become obsolete to a critical level [3]. There are approximately 1.4 million street lighting poles in the Czech Republic and its total value is approximately CZK 98 billion. The annual investment in the entire system is approximately CZK 1.75 billion. According to the Society for the Development of Street Lighting, the estimated lifetime of street lighting is approximately 40 years. For the luminaire itself, it is less than half [4,6].

The new generation of LED luminaires is characterized by high efficiency in operation at maximum output as well as in operation at lower output when the illumination intensity is regulated to the desired level [3,7,8]. The first generation of these luminaires had a low power factor (PF) in the range of 0.4 to 0.6. A low power factor value causes the electrical device to consume more current at the same output compared to a situation when the power factor was closer to value 1 [9,10]. The negative impact of this fact is a higher loss in the distribution of electricity compared to a state when the power factor equals or approximates 1. In the next generation, LED luminaires were equipped with a Power Factor Correction (PFC) circuit that increased the power factor to values ranging from 0.8 to 0.95. There are two types of PFC circuits: passive and active [7,11,12]. Passive PFCs are used for equipment of up to 100 Watts. The power factor correction method is based on the use of a passive low-pass filter to suppress higher harmonic components of the current. The filter consists of a serial connection of the LC circuit. Active PFCs are used for circuits with power consumption higher than 100 W, and the principle is based on a suitable method of controlling the circuit of the power supply being switched. The control circuit measures the input voltage and the current, and subsequently adjusts the duty cycle and the switching frequency so that the current and the voltage were phase-shifted as little as possible. Active PFCs not only improve PF, but they also decrease the harmonic distortion of the THD current. However, this does not mean that using an active PFC leads to a sinusoidal shape. Three-phase PFC problematics is further explored by Kolar et al. [13] and Friedli et al. [14]. During the simultaneous operation of various appliances, the power quality (PQ) tends to deteriorate significantly. Therefore, there is still room for compensation of higher harmonics currents, which would lead to improvements in PQ.

Another way of saving money for street lighting operation is to optimize the performance and usability of this lighting [6,15]. This can be achieved using various control methods. For example, if, at a specific time, the natural ambient light is of sufficient intensity, the street lighting will not illuminate at 100% power, but only at 60%. Another possibility is to illuminate street areas at a lower level at night and to detect movement of people or vehicles by means of this area using motion sensors [16]. Lighting control responds to movement by increasing the lighting level during the movement. The benefit is that the luminaires only illuminate at 100% power when needed and not all night long. LED luminaires are a suitable technology for the operation of SMART lighting, as they allow easy regulation of light intensity.

With LED intensity control, there is a problem associated with the behavior of the LED power supplies, which are usually designed and optimized for operation at 100% of the possible power and not, for example, for half power. When operating at lower power, the resulting power consumption of the lighting system decreases, but, on the other hand, the extent of the higher harmonic components of the current is increased, as the power circuits do not operate in an optimum mode. Higher harmonic components of the current subsequently cause a deterioration in the voltage quality due to distribution network impedance, which can cause a number of negative consequences. Negative impacts of poor voltage quality are, for example, shortening the lifetime of some electrical equipment, malfunction of protections, poor functionality of Mass Remote Control (MRC) receivers, or electrical motor overheating [10]. In some cases, it is necessary to choose a compromise between the energy saved and the degree of deterioration in the quality of electricity.

The quality of electric energy can be improved by means of filtering devices. According to the basic classification, filtration is divided into passive and active filtration. Passive filtration means that a filter for a particular harmonic component having fixed parameters is inserted into the circuit [17]. This type of filter cannot dynamically respond to possible changes in the interference spectrum. The second type of filter used is the active filter. The active filter is a filter controlled by the control system, and thus it allows dynamic changes in the filter properties depending on the current interference status. Active filters are divided into series active and shunt active power classes. The serial voltage active filter allows correction of the voltage amplitude, compensation of drops and peaks, removal of higher harmonic components, and achieving voltage symmetry. This filter does not allow working with the current amplitude.

The Shunt Active Power Filter (SAPF) is essentially a controlled current generator that injects the current into the part of the electrical circuit being compensated in parallel, via the coupler [18]. The control unit contained in the filter uses a current sensor to measure the instantaneous current waveform that is processed in the control unit by a suitable algorithm. The correction signal, which is amplified by the power section of the filter and produces a compensating current, is obtained by calculation. The compensating current is injected into the network and the compensating current is added to the network current. This results in significant suppression of the higher harmonic components in the resulting total current. Further, by generating a positive sequence and negative sequence, an unsymmetrical 3-phase load can be corrected so as to appear symmetrical by using a filter. Both types of filters can be combined into one comprehensive unit. The serial part of the filter improves voltage, while the current part of the filter corrects the disturbing effects of the load connected.

The authors of the article focus mainly on the research of control of SAPF [18]. The main objective of the research is modern control methods that are able to dynamically react to changes in the interference spectrum. The area of modern SMART street lighting systems is very dynamic in terms of electricity consumption and it is necessary that the filtration is designed for this fact in consideration. Therefore, static filtration cannot be used because it cannot effectively cover all of the interference variants that occur. Conversely, active filtration can respond to changing interference and adjust filter settings for optimal interference suppression using appropriate algorithms.

Compensation of higher harmonic components of the current is performed mainly because they cause a change in voltage shape or impair the voltage quality with a number of negative consequences. Examples of negative impacts may include higher load on phase conductors, unexpected load on the neutral conductor, overheating of transformers and electrical motors, possible destruction of compensating capacitors, or reduction of the service life of electrical equipment.

Although LEDs and SMART elements in street lighting do not generate higher harmonic components of significant performance as individual devices, it is necessary to take into account the deployment of thousands of SMART elements within one city, because each lighting pole can contain, in addition to the light source, the SMART elements stated further in the text. In summary, this is a potentially large source of interference. SMART elements are primarily camera systems, measuring systems, charging devices for mobile electronics and e-mobility, security systems, information systems, etc. Each of these elements contains a switching power supply, and the switching power supplies are the biggest cause of the higher harmonic components of the current [3].

It follows from the above that the issue of network interference in modern street lighting is a very topical matter. In response to these problems, the BROADBAND${}^{\mathrm{LIGHT}}$ test polygon was built in the parking lot next to the Faculty of Electrical Engineering and Computer Science at the VSB–Technical University of Ostrava Campus [16,19,20,21]. The test polygon allows testing of new street lighting technologies; measuring their impact on the power quality in various operating states [22]; and, in the future, also testing active filtration [23].

Furthermore, various methodologies of SAPF control using adaptive algorithms are described in this work. Then, there is a text concerning the newly created test polygon for the needs of the testing LED light sources and SMART elements and their influence on the quality of electric energy. The next chapter describes the experiments carried out in the polygon and their methodology. Both the HW part of the polygon control and monitoring as well as the results for the individual types of adaptive filtering algorithms being examined are described. Next, there is a description of the implementation of the Least Mean Squares (LMS) algorithm on Field Programmable Gate Array (FPGA) and real experiments with network interference filtration. The last chapter contain a discussion on the results achieved and also to the outline of the future work.

## 2. Methods

**Figure 1:**
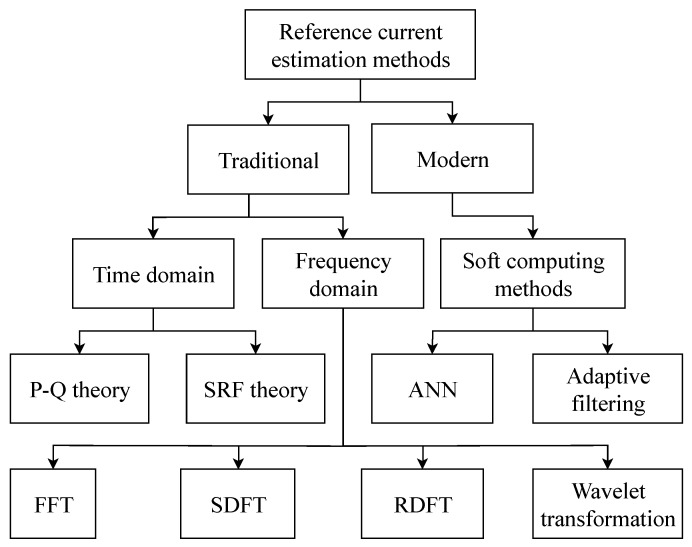
Schematic diagram of current estimation methods.

Figure 1 shows the basic distribution of SAPF algorithms for harmonic component extraction, which are generally divided into three basic groups: time-domain [24,25,26], frequency-domain [27,28,29], and soft computing methods [30,31].

Time-domain algorithms for harmonic component extraction calculate the reference current signal in two different ways: The first way is to use the p–q methods [24,25,32,33,34], Unity Power Factor (UPF) [26,35], Perfect Harmonic Cancellation (PHC) [26,36], or Synchronous Detection Method (SDM) [36,37,38], which operate with instantaneous powers in a harmonic distorted power system. The second way is comprises use of the Synchronous Reference Frame Method (SRF) [25,37] or Id-Iq [26,39,40], which works with instantaneous current values. These methods have the lowest computational demands and have already been described by the team of authors in the publication on [41].

Frequency-domain algorithms for harmonic component extraction include Discrete Fourier Transform (DFT) [42], Fast Fourier transform (FFT) [43,44,45,46], Recursive Discrete Fourier Transform (RDFT), Kalman filter, and others [47]. These algorithms are characterized by high computational complexity and, in transient processes, a method error occurs because the Fourier transform requires an entire period or an integral multiple for reference computation.

Soft computing methods are suitable for the extraction of harmonic components, especially in a time-varying environment, as these methods can adapt to the environment. The main representatives of these methods are adaptive filtering [48,49,50,51], fuzzy logic [34,52,53,54]; artificial neural networks [30,55,56,57,58] and genetic algorithms [59,60,61] or, possibly, their combinations, e.g., the Neuro-Fuzzy approach [34,62].

In this study, the following methods were used for suppressing higher harmonic components in the electrical network; Clarke transformation, notch adaptive algorithms least mean squares (LMS) filter, and recursive least squares (RLS) filter. Clarke transformation is used to obtain a reference signal x(n) for the adaptive algorithm. Using the adaptive algorithm, we obtain a three-phase compensating current for the converter that generates the compensating current.

### 2.1. Clarke Transformation

**Figure 2:**
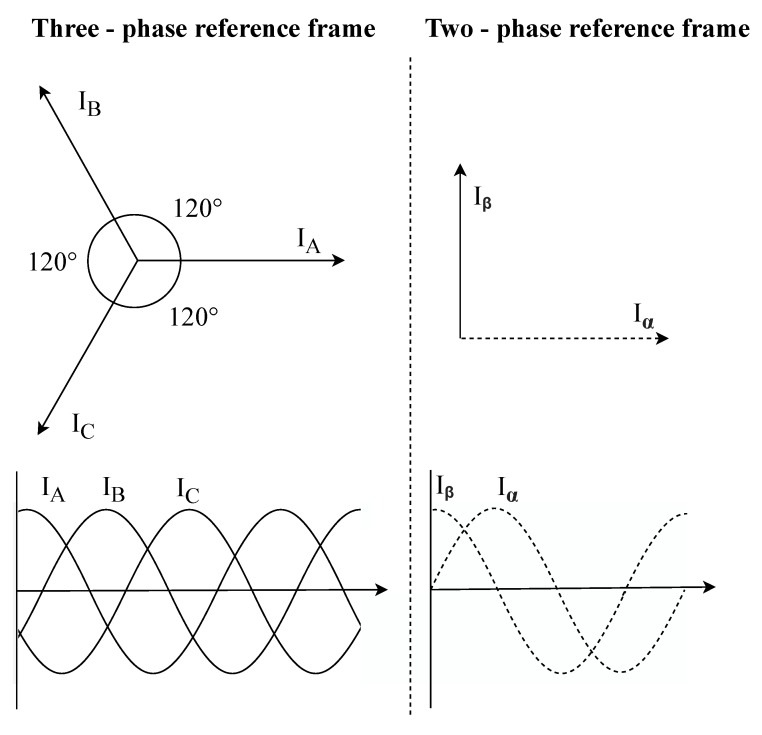
Clarke transformation coordinates.

This transformation converts three-phase coordinates a-b-c to two-phase coordinates α-β-0, as shown in Figure 2. The current waveforms obtained by Clarke Transformation serve as a reference for adaptive filtering [63,64]. Transformation itself can be seen in Equation (1) for voltage signals and Equation (2) for current signals.

(1) $$\left[\begin{array}{c} U_{0}\\ U_{\alpha }\\ U_{\beta } \end{array}\right]=\sqrt{\frac{2}{3}}\cdot \left[\begin{array}{ccc} \frac{1}{\sqrt{2}} & \frac{1}{\sqrt{2}} & \frac{1}{\sqrt{2}}\\ 1 & -\frac{1}{2} & -\frac{1}{2}\\ 0 & \frac{\sqrt{3}}{2} & -\frac{\sqrt{3}}{2} \end{array}\right]\cdot \left[\begin{array}{c} U_{a}\\ U_{b}\\ U_{c} \end{array}\right],$$

(2) $$\left[\begin{array}{c} I_{0}\\ I_{\alpha }\\ I_{\beta } \end{array}\right]=\sqrt{\frac{2}{3}}\cdot \left[\begin{array}{ccc} \frac{1}{\sqrt{2}} & \frac{1}{\sqrt{2}} & \frac{1}{\sqrt{2}}\\ 1 & -\frac{1}{2} & -\frac{1}{2}\\ 0 & \frac{\sqrt{3}}{2} & -\frac{\sqrt{3}}{2} \end{array}\right]\cdot \left[\begin{array}{c} I_{a}\\ I_{b}\\ I_{c} \end{array}\right].$$

### 2.2. Notch Adaptive Filter

**Figure 3:**
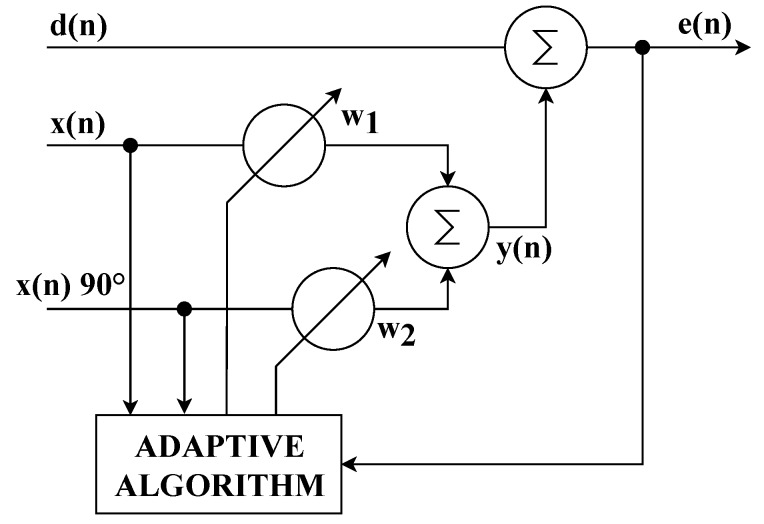
Block diagram of the notch adaptive filter.

Figure 3 illustrates the principle of the Notch Adaptive Filter. This filter is able to process multiple reference input signals from different sources. One transverse Finite Impulse Response (FIR) filter weights vector coefficient ($w_{i}$) is needed for each reference input signal. In this study, two $w_{i}$ coefficients are used, and the filter is used to process two input signals that are phase-shifted by 90${}^{\circ }$. The notch adaptive filtering procedure is similar to the classical adaptive filtering procedure [65,66,67].

### 2.3. Notch Adaptive LMS Algorithm

The LMS algorithm is an essential representative of stochastic gradient adaptation. Stochastic gradient descent is one of the most commonly used iterative methods for optimizing differentiable objective functions, due to its ability to effectively accelerate the learning process. This method is named stochastic as the samples are selected randomly and not as one group (as in the standard descent gradient) or in the order in which they appear in the training set. Each LMS algorithm iteration requires three different steps to be performed in the given order. Notch LMS is a modification of the classical LMS algorithm for the notch adaptive filter structure. As with the classical LMS, the notch LMS algorithm requires three different steps to be performed in the order given [65,68].

- Calculation of the FIR filter output value:
  (3) $$y\left(n\right)=\vec{x}\left(n\right)\cdot {\vec{w}}_{1}^{T}\left(n\right)-{\vec{x}}_{90^{\circ }}\left(n\right)\cdot {\vec{w}}_{2}^{T}\left(n\right).$$
- Calculation of the estimated e(n) error signal:
  (4) e(n)=d(n)−y(n).
- Finally, the vector weight values ${\vec{w}}_{1}\left(n\right)$ and the ${\vec{w}}_{2}\left(n\right)$ corresponding to the FIR filter values are updated with respect to the following iteration,
  (5) $${\vec{w}}_{1}\left(n+1\right)={\vec{w}}_{1}\left(n\right)+2\mu e\left(n\right)\vec{x}\left(n\right).$$
  (6) $${\vec{w}}_{2}\left(n+1\right)={\vec{w}}_{2}\left(n\right)+2\mu e\left(n\right){\vec{x}}_{90^{\circ }}\left(n\right).$$

Note that the choice of the μ convergence constant (step size) has a major influence on the function of the filter. Too large a value of the step size results in loss of stability and also inaccurate filtration. Conversely, a low step size causes an increase in computation time and augments computation demands.

### 2.4. Notch Adaptive RLS algorithm

Unlike the LMS algorithm inputs that are considered stochastic, the Recursive Least Squares (RLS)-based algorithms are considered deterministic. Implementation of the RLS algorithm consists of calculation of gain vector k(n), error estimation e(n), vector coefficients w(n), and matrix $P\left(n\right)=R_{\mathrm{XX}}^{-1}\left(n\right)$. Notch RLS is a modification of the classical RLS algorithm for the notch structure. To implement the RLS algorithm, the following steps must be performed in the following order [65,69].

- The filter output is calculated using filter weights from the previous iteration and the current input vector:
  (7) $$y\left(n\right)={\vec{w}}_{1}^{T}\left(n\right)\vec{x}\left(n\right)+{\vec{w}}_{2}^{T}\left(n\right){\vec{x}}_{90^{\circ }}\left(n\right).$$
- The mean gain vector is calculated using the following equation.
  (8) $$k_{1}\left(n\right)=\frac{P\left(n-1\right)\vec{x}\left(n\right)}{\lambda +{\vec{x}}^{T}\left(n\right)P\left(n-1\right)\vec{x}\left(n\right)}.$$
  (9) $$k_{2}\left(n\right)=\frac{P_{90^{\circ }}\left(n-1\right){\vec{x}}_{90^{\circ }}\left(n\right)}{\lambda +{\vec{x}}_{90^{\circ }}^{T}\left(n\right)P_{90^{\circ }}\left(n-1\right){\vec{x}}_{90^{\circ }}\left(n\right)}.$$
- The value of the estimated error is calculated according to the following equation.
  (10) $$e\left(n\right)=d\left(n\right)-{\vec{w}}_{1}^{T}\left(n-1\right)\vec{x}\left(n\right)+{\vec{w}}_{2}^{T}\left(n-1\right){\vec{x}}_{90^{\circ }}\left(n\right).$$
- The weights vector is updated using the following equation.
  (11) $${\vec{w}}_{1}\left(n\right)={\vec{w}}_{1}\left(n-1\right)+e\left(n\right)k_{1}\left(n\right).$$
  (12) $${\vec{w}}_{2}\left(n\right)={\vec{w}}_{2}\left(n-1\right)+e\left(n\right)k_{2}\left(n\right).$$
- The inverse matrix is calculated using the following equation.
  (13) $$P\left(n\right)=\lambda^{-1}P\left(n-1\right)-\lambda^{-1}k\left(n\right){\vec{x}}^{T}\left(n\right)P\left(n-1\right).$$
  (14) $$P_{90^{\circ }}\left(n\right)=\lambda^{-1}P_{90^{\circ }}\left(n-1\right)-\lambda^{-1}k_{90^{\circ }}\left(n\right){\vec{x}}_{90^{\circ }}^{T}\left(n\right)P_{90^{\circ }}\left(n-1\right).$$

The RLS algorithm functionality is fundamentally influenced by the choice of the forgetting factor λ, having the values of λ∈(0,1]. If λ=1, then we are talking about an estimate without forgetting. Due to the shape of $\lambda_{n}-i$, we can talk about weighting. The input values of the individual signals before i=1 are considered as zero; the values from the last set of values are significant. Thus, the matrix or the vector of mutual correlations are weighted by the previous autocorrelation, adding the correction for the relevant values of the autocorrelation of the matrix or the vector of mutual correlations, see Equations (8) and (9). In practical implementation, we usually consider from λ=0.98 to λ=1. For small values of the coefficient λ, there is little contribution of the previous samples to the covariance matrix. This makes the filter more sensitive to current samples.

### 2.5. Three-Phase Shunt Active Power Filter

**Figure 4:**
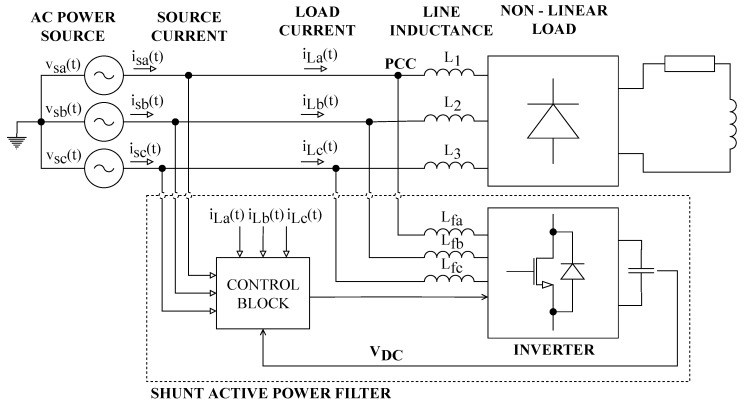
Block diagram of the system for compensation of higher harmonic components using Shunt Active Power Filter (SAPF).

Figure 4 shows a power system configuration containing SAPF. The purpose of SAPF is to compensate for the higher harmonic components contained in the power supply system due to nonlinear loads. The compensation itself occurs by SAPF delivering harmonic components generated by nonlinear loads to the Point of Common Coupling (PCC), but in the opposite phase. The SAPF can also compensate for idle power without any resonance. The SAPF is connected to the power system via an output filter to minimize switching ripples. Further information on SAPF can be found in [54,61,70,71,72].

The SAPF structure consists of two main parts: a control block and a voltage source inverter. The control block generally contains four main control algorithms: (1) harmonic extraction, (2) DC-link capacitor voltage regulation, (3) current control, and (4) synchronizer algorithms. The voltage source inverter contains six IGBT semiconductor switches in a three-phase system, which are connected in a bridge circuit [70].

## 3. SMART BROADBAND${}^{\mathrm{LIGHT}}$ Test Polygon

**Figure 5:**
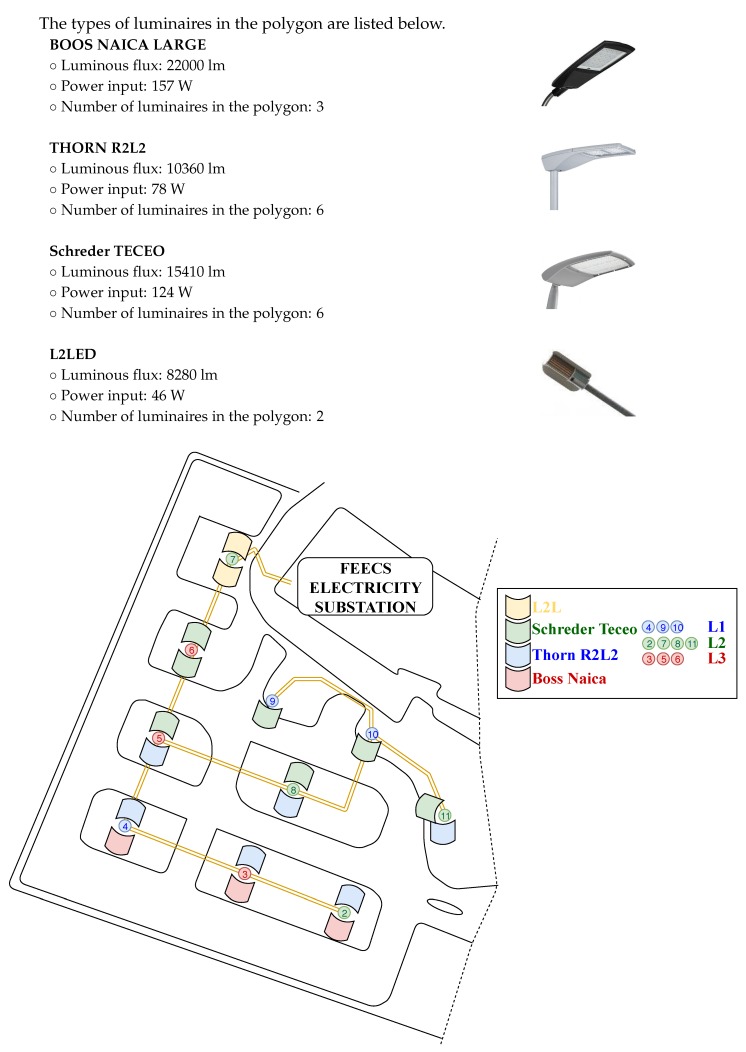
SMART car park plan.

**Figure 6:**
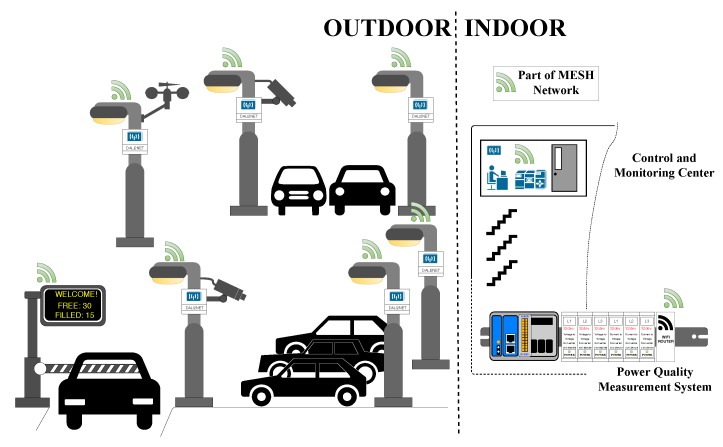
A simplified idea diagram of the SMART car park components deployed.

**Figure 7:**
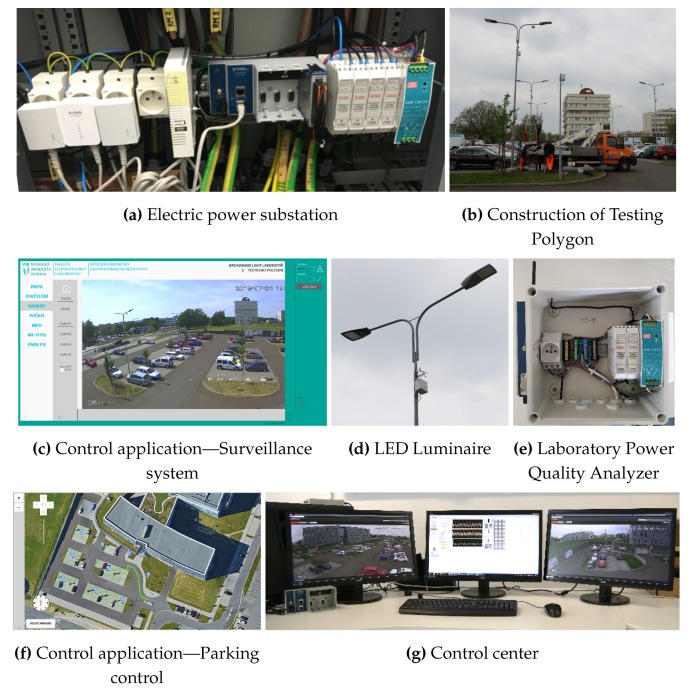
Photographs of the testing polygon.

The SMART BROADBAND${}^{\mathrm{LIGHT}}$ test polygon is situated in the parking lot of the Faculty of Electrical Engineering and Computer Science at the VSB–Technical University of Ostrava Campus [73]. It currently contains 10 pylons for mounting of various equipment. The pylons are fitted with homologated luminaires in accordance with the applicable standards for the operation of street lighting in the Czech Republic. In total, they are fitted with four types of luminaires, which are conventional, and one type of experimental luminaire (L2L) that is being developed. Each of the pylons is also equipped with its own switchboard, in which a Wi-Fi router + various types of SMART sensors and SMART elements are installed. The individual pylons communicate with each other via a wireless network and, together, they form a Full-Mesh topology network. This network is controlled remotely from the lab where the network server is located as well as several PCs that can communicate with and manage the car park infrastructure. Each pylon forms a separate unit and can be controlled independently of the others, so that individual luminaires can be controlled independently. The parking lot plan can be seen in Figure 5. A simplified idea diagram of the SMART car park can be seen in Figure 6. In Figure 7, various photos from testing polygon are shown.

### 3.1. Luminaires in the Polygon

Currently, the polygon is fitted with four types of luminaires. All luminaires use LED light sources and there are 17 light sources altogether. Switching of the luminaires is performed by the DALI communication interface, which is converted to the Ethernet interface using a DALI2ETH converter. Each pylon is equipped with its own switchboard, which contains a Wi-Fi router, a 24VDC power supply, a DALI-Ethernet converter, and also other SMART technologies (a camera system, an Ethernet speaker, information boards, weather stations, etc.). Moreover, three switchboards use information transmission via the Power Line Communication (PLC) technology, where each of these luminaires is connected to a different phase of the electrical network.

### 3.2. System for Measurement and Evaluation of Electrical Power Quality

**Figure 8:**
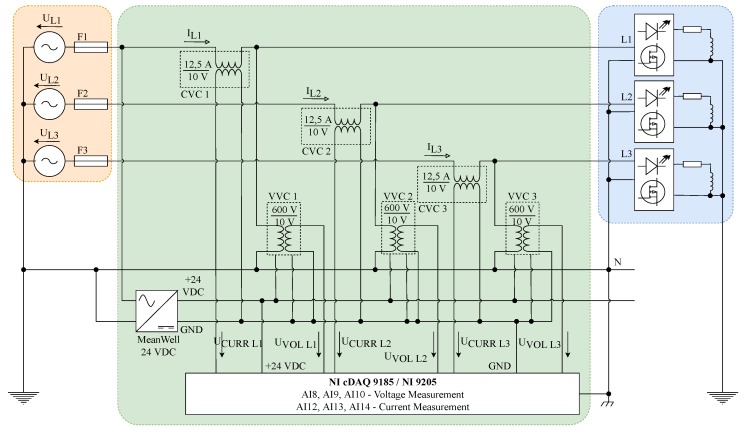
Measurement system wiring diagram.

The system for measurement and evaluation of electric power quality designed according to standards IEC 61000-4-30 [74] and IEC EN 50160 [75] is also an integral part of the polygon system. The system is based on the modular measuring system equipped with National Instruments (NI) data acquisition HW. Special signal conditioning modules are used to measure three voltages of 230VAC and three currents. Signal conditioning modules outputs are connected to data acquisition HW. System wiring diagram can be seen in Figure 8.

### 3.3. Data Acquisition HW

**Figure 9:**
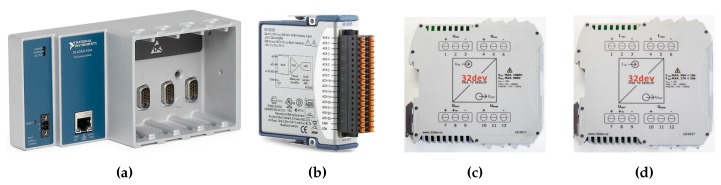
Power quality (PQ) system components: (**a**) NI cDAQ 9185 4-slot chassis, (**b**) NI-9205 data acquisition module, (**c**) voltage converter, and (**d**) current converter

The measurement system is based on the NI cDAQ-9185 4-slot chassis, which provides digitized data via Ethernet (Figure 9a). One slot in the chassis is fitted with the NI-9205 (Figure 9b) 32-input data acquisition module containing a 16-bit AD converter. The advantage of this concept of modular measuring HW is the simple extension of the system by additional HW modules and their use by modification of the software for operating this device. The cDAQ-9185 chassis is accessed by the software developed using NI-DAQ libraries. The data is read by means of a program implemented in LabVIEW on a PC in the control laboratory.

### 3.4. Signal Conditioning Modules for Voltage and Current Signals

The signal conditioning modules are designed for use in the field of electric power quality measurement. The voltage converter (Figure 9c) has 2 input ranges: 600 V and 300 V. The current converter (Figure 9d) has 2 input ranges: 25 A and 12.5 A. The output signal has an amplitude of ±10 V. The modules are characterized by minimal harmonic distortion and high linearity of conversion. The frequency range of the converters is 100 kHz, with the option to activate a low-pass filter with a cut-off frequency of 2.5 kHz, which corresponds to the 50th harmonic component of the supply voltage [76,77].

The measuring HW is located in the substation of the faculty building from where the data is transmitted to the control laboratory via LAN. The electrical diagram of the system for measurement and evaluation of electrical power quality can be seen in Figure 8.

### 3.5. Server

The control server of the entire system is located within the control laboratory. The server runs on the Debian Linux distribution, which guarantees stability, easy maintenance, and is very popular in server installations. The server has both the MQTT (Message Queuing Telemetry Transport) broker control function and the functions of a web server as the database of all data that will pass through the test polygon [16,73]. The database is built on PostgreSQL, which is an open source object-relational database system. The web server is created in Apache HTTP Server and Daphne 2.1.0. The application framework for polygon handling runs on the Django platform.

### 3.6. Control Applications

**Figure 10:**
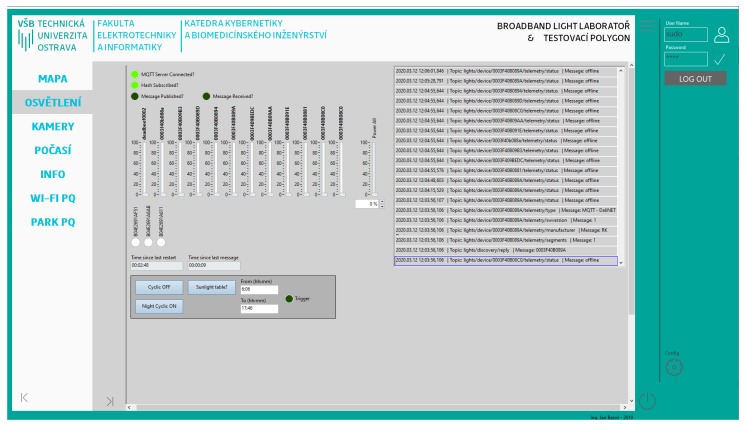
Front panel of control application of testing polygon.

The LabVIEW development environment was chosen for system integration of all systems into one unit. LabVIEW is extremely suitable for fast prototyping and excellent support of data acquisition. For the experiments, an application that can perform a sequence of commands with the test polygon and continuously records the voltage and current flowing in the test polygon electrical network has been designed. For simplicity reasons, we will call the sequence of commands a ”scenario”. The front panel of the control application written in LabVIEW can be seen in Figure 10.

First, the application loads the scenario, which is a specifically formatted .CSV file (example of formatting can be seen on Table 1). This scenario includes relative execution time of the command, the command itself, and the performance of each luminaire. Furthermore, the scenario contains the necessary delay time after the command is executed. The delay before the next command is necessary, because after sending the command, the polygon infrastructure needs up to 2 secs to execute the command, depending on the command type and the number of luminaires included in the command. Once all the commands are executed, a command to completely turn off the luminaires in the test polygon is sent and the process for operating the scenario execution is completed.

In parallel with the polygon control process, there are 3 other processes running: (1) measurement using the DAQ system, (2) recording of the data measured, and (3) operation of the MQTT communication.

MQTT stands for Message Queuing Telemetry Transport. It is a simple standardized protocol that is used to exchange messages between devices. Its most common use is in Internet of Things (IoT) applications. The protocol is designed to be used in large networks with a low data stream. It is adapted to use the smallest possible data streams. It is, therefore, suitable for use in situations where it is necessary to periodically gather data or the operation of the data network is somehow limited, e.g., slow network speed, high latency, or frequent network outages [73].

**Figure 11:**
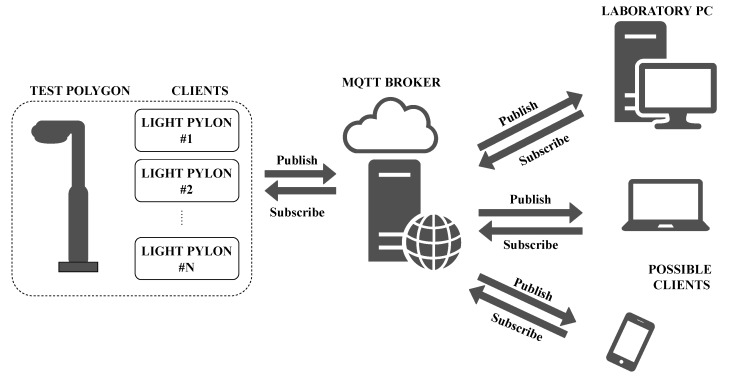
Diagram of possible MQTT network.

The principle that it operates an is that a device that acts as a client sends messages to, and receives messages from, the server. The filtration of unwanted messages is performed in such a way that the device subscribes only to those subjects on which it is written and ignores others. The subject is called the *topic*. The part of the message containing the data is called the *Message*. Each message contains these two items. The server also serves as a data concentrator for all messages from all clients connected. It is called the MQTT broker. Each device can work in three various modes: either it can only receive messages, send messages, or work in both modes. An idea diagram of a currently used MQTT network on a test polygon can be seen in Figure 11.

Simultaneously with the start of the application, the measurement of electrical quantities by means of a data collection system is commenced. As part of the initialization, the NI cDAQ-9185 measurement chassis is connected. The measurement channels are then created and the sampling rate is set. Furthermore, the measurement and the cyclic continuous reading of the data from the measuring card start. Next, the data is sent into the process for writing data to a file via the FIFO queue. The program runs until the scenario processing announces the end of the scenario; then, the measurement is terminated and the measurement chassis is disconnected.

At the start of the measurement, the data writing process first creates a TDMS file with a unique name and then begins to receive the continuously measured data by reading the queue. It cyclically saves data in each waveform to the TDMS file. The application does not store data in any buffer before saving either; memory leak is therefore avoided. The whole application runs until the ”end of scenario” message arrives. Then, the last data are saved and the file is closed.

In the case of MQTT communication, the connection to the MQTT broker will first be established using the IP address and port 1883, which is specified in the MQTT v3.1 standard [73]. Subsequently, there is a wait time and FIFO messages containing commands from the scenario are received. These commands are then converted to the specific MQTT message. MQTT topic *device/lights/”MAC address of the second pole”/power/set* with MQTT message 100.00 corresponds to scenario with index 3 from Table 1.

**Figure 12:**
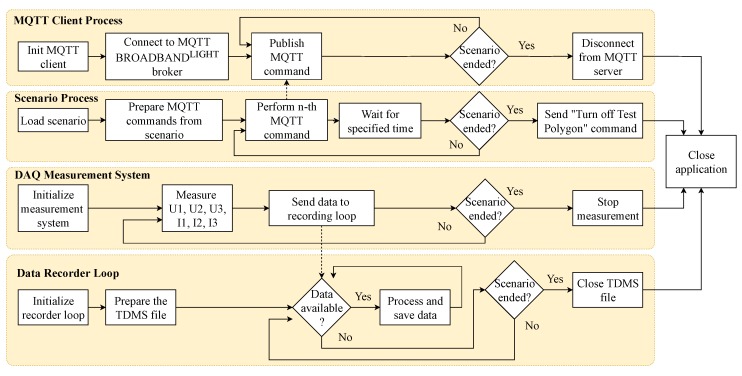
Algorithm for experiment execution.

As the luminaires do not instantly respond to the received command, there is a wait time after each command, the length of which was chosen experimentally. The measurement revealed that a wait time of approximately 2 secs is safe enough for sending one command. Furthermore, because of the possibility of network congestion by the camera system running concurrently with the measurement, 1 sec was added to the wait time. Thus, a wait time of 3 secs is selected for one command. MQTT messages are sent to all ten luminaires when special commands *Full OFF* or *Full ON* are entered. Here, a wait time of 10 secs is selected at the beginning so that the data measured immediately shows where the polygon was switched off. From this starting point, further computational experiments were performed. After the end of the scenario is announced, the connection to the MQTT server is terminated correctly. Figure 12 shows the simplified scheme of the previously mentioned functionalities.

## 4. Experiments

**Figure 13:**
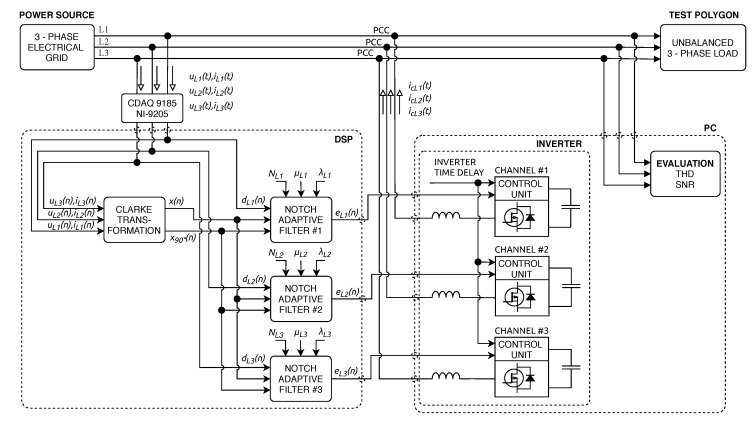
Simplified schematic diagram of the conducted experiment with simulated inverter.

**Figure 14:**
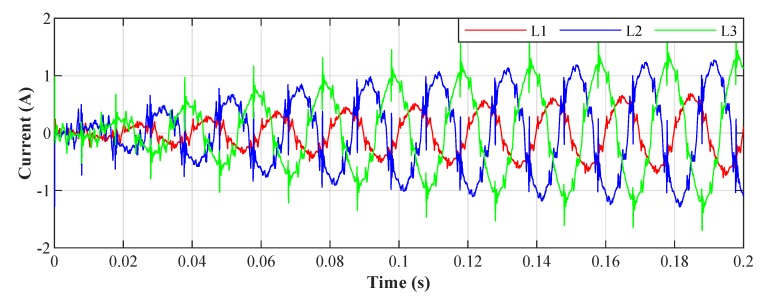
Idle run of test polygon; current waveforms after applying the least mean squares (LMS) algorithm.

**Figure 15:**
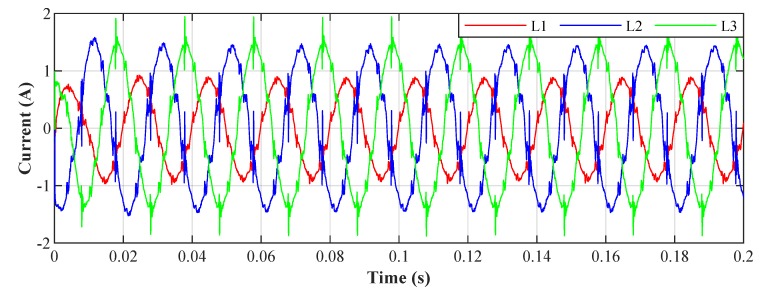
Idle run of test polygon; current waveforms after applying the recursive least squares (RLS) algorithm.

**Figure 16:**
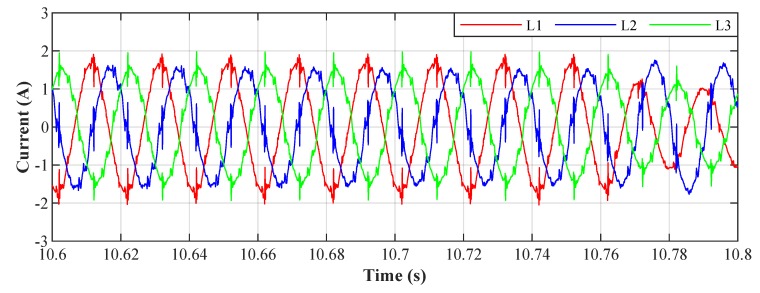
Switched on test polygon; current waveforms after applying the LMS algorithm.

**Figure 17:**
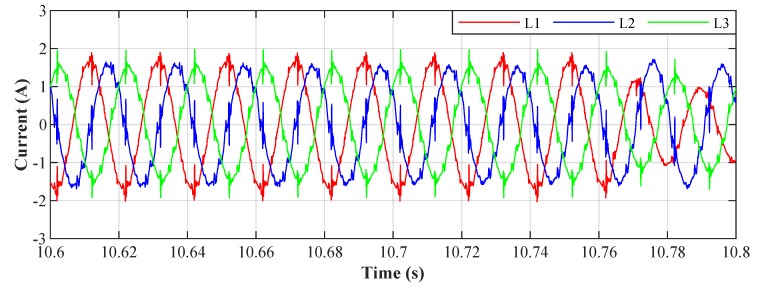
Switched on test polygon; current waveforms after applying the RLS algorithm.

To verify the theoretical assumptions of SAPF, we simulated the extraction of compensating currents $i_{cL1}\left(t\right)$, $i_{cL2}\left(t\right)$, and $i_{cL3}\left(t\right)$. Figure 13 shows a diagram illustrating the experiment with the simulated inverter. Currents $i_{L1}\left(t\right)$, $i_{L2}\left(t\right)$, and $i_{L3}\left(t\right)$ and voltages $u_{L1}\left(t\right)$, $u_{L2}\left(t\right)$, and $u_{L3}\left(t\right)$ are measured using NI cDAQ-9185 with NI-9205 module. Subsequently, the whole experiment is performed on a PC and the compensating current is obtained. The three-phase signal is first converted using a Clarke transform of x(n) and $x_{90^{\circ }}\left(n\right)$. These signals are then used as the primary inputs of each *Notch Adaptive Filter*, where the individual phases of the measured signal are used as reference inputs d(n). The error signal e(n) is then used as the primary input of in the simulated inverter. Figure 14, Figure 15, Figure 16 and Figure 17 show the results of these simulations. It can be noticed that the noise in the output signal was not completely suppressed. This is due to the artificial delay that has been introduced and corresponds to the latency of the real inverter. The unbalanced three-phase load indicates the testing polygon itself. To provide better conditions for SAPF testing, the polygon was deliberately designed as an unbalanced load.

### 4.1. Comparison of Various Luminaries

**Figure 18:**
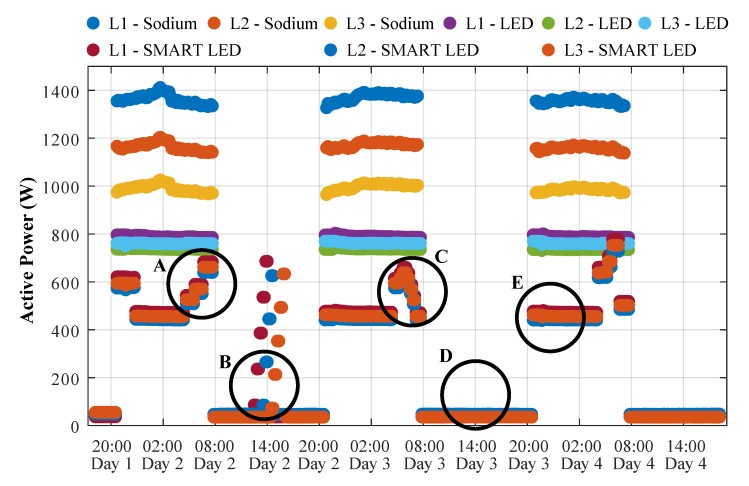
Comparison of various types of luminaries.

Figure 18 shows the general course of the four-day consumption measurement on the previously described testing polygon. A total of 3 public lighting technologies are compared, namely, sodium-based, LED-based, and SMART LEDs. SMART LEDs are operated 24/7, whereas the other lighting technologies are only turned on when necessary, mainly during night and morning hours. The measured waveforms show that the SMART LEDs consume approximately 45W; however, when compared to conventional uncontrolled LED technology, the SMART LEDs operation was only maintained at 60% of total power to further save energy. In addition to the standard operation of the testing polygon—night lighting—various other scenarios were tested as well (letters corresponds with Figure 18):

- Because of the morning arrivals of people to work, as the polygon fills up, the individual lighting poles responds and light up to almost full power.
- Daily testing of the various components of the testing polygon.
- A similar scenario as A), but, in addition, the rising sun was taken into account—with gradually higher ambient light intensity, there was no need to illuminate at higher power.
- The consumption of SMART elements on the polygon is approximately 45W.
- In the case of no recorded event (passing car, pedestrian, etc.), SMART lighting operation is maintained at 60% of total power.

### 4.2. Filter Settings Optimization

**Figure 19:**
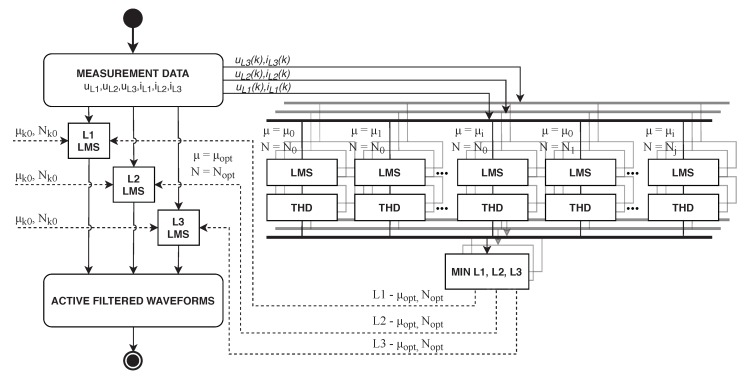
Diagram of optimization experiment of LMS algorithm setting.

To determine the optimal settings of the LMS algorithm, an optimization algorithm was used, in which the filter length *N* and step size μ were gradually increased. The filter length was increased by ΔN=1, whereas the step size was increased by $\Delta \mu =1\times 10^{-7}$. The final values were $N_{MAX}=20$ a $\mu_{MAX}=10\times 10^{-5}$. Subsequently, the value of THD was thoroughly examined and its global maximum was sought. The settings coordinates for this specific $THD_{min}$ were $N_{opt}$ and $\mu_{opt}$. The optimization was carried out separately for each individual phase. The block scheme of this experiment can be seen in Figure 19. A similar approach was used in [78].

### 4.3. Laboratory Experiment

**Figure 20:**
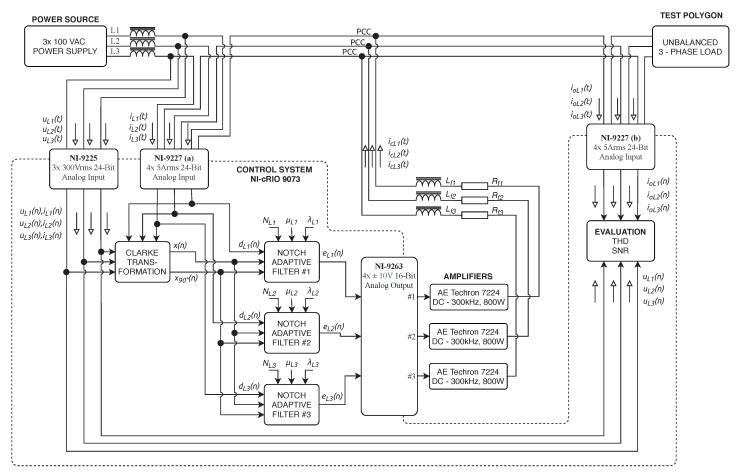
Schematic diagram of realized laboratory experiment with AE-Techron 7224 amplifiers used as inverters.

**Figure 21:**
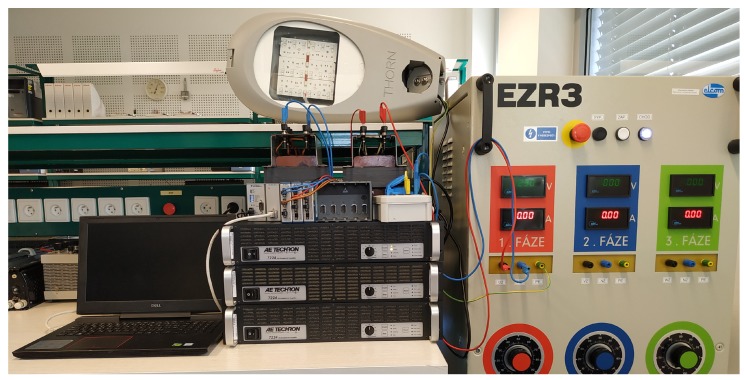
Realized laboratory experiment with AE-Techron 7224 amplifiers used as inverters.

In addition to the previously described experiment, a single-phase load variant was performed as well. The simulated converter was replaced by a real solution, consisting of a control unit built on the NI cRIO-9073 and an AE-TECHRON 7224 power amplifier. The control unit measures voltage signals through the NI-9225, whereas the current is measured by NI-9227 twice. The compensation current is generated through the analog output module NI-9263, amplified by AE-TECHRON 7224 amplifier, and coupled to the compensated circuit via the R-L coupling circuit. The frequency range of the amplifier is DC 300 kHz, the maximum voltage is 158 V, and the maximum current is 50 A. The supply network was replaced by a controllable AC source, and the short-circuit impedance was increased by series inductance. The block diagram of the experimental setup is shown in Figure 20, and the photograph of the whole experiment can be seen in Figure 21.

### 4.4. FPGA Experiment

**Figure 22:**
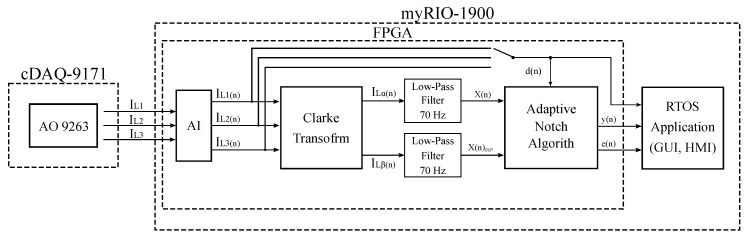
Diagram of the conducted experiment.

To achieve the highest possible filtration quality, it was necessary to minimize the reaction time between data acquisition and the subsequent reaction of the inverter, i.e., by injecting the compensating current back into the load. For this reason, the FPGA was used for the implementation because it provides the possibility of almost parallel data processing. Figure 22 shows a block diagram of the experiment with FPGA described in Section 4.6.

**Figure 23:**
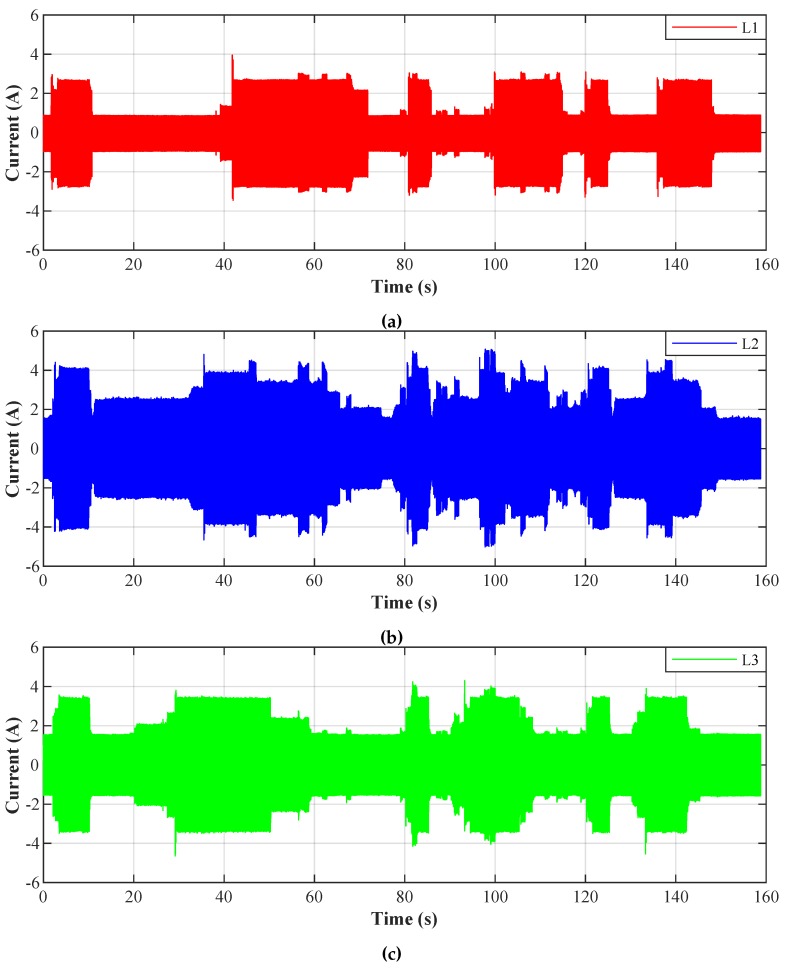
Current waveform envelopes of the test polygon. (**a**) L1—phase 1, (**b**) L2—phase 2, and (**c**) L3—phase 3.

Figure 23 shows the current waveform envelopes of the scenario developed, which are described in Section 3. The LED luminaires were switched on and off for 160 seconds according to the predefined scenario. The result is a varying three-phase unbalanced load with varying levels of higher harmonic components in the electrical network.

On these real waveforms it is demonstrated how the tested adaptive filter algorithm adapts to different loads over time. In the simulated experiments, no real active filter was used to compensate for higher harmonic components. To respect the properties of the real active filter, a 100 μs delay was applied in the simulations, which simulates the latency of the real converter to which the adaptive algorithms will be applied.

### 4.5. Results

**Figure 24:**
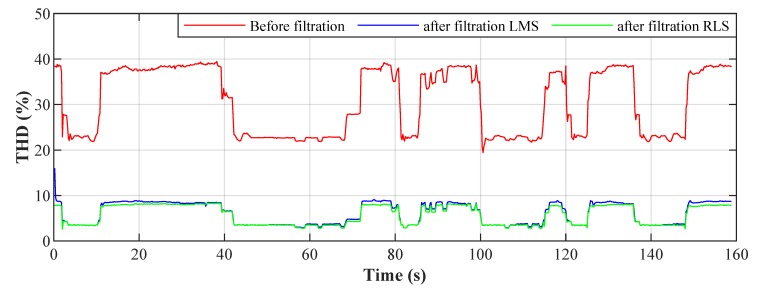
Total Harmonic Distortion (THD) values at intervals of 200 ms.

Figure 24 shows the Total Harmonic Distortion (THD) of the current on the L1 phase without filtration and with filtration applied. The THD values were calculated from time windows Tw = 200 ms. The changes in THD over time are caused by switching the LED luminaires on and off. The graph shows that it is possible to achieve THD reduction, and thus suppression of the higher harmonic components, by applying adaptive filtration and that both of the adaptive algorithms used achieve similar results.

**Figure 25:**
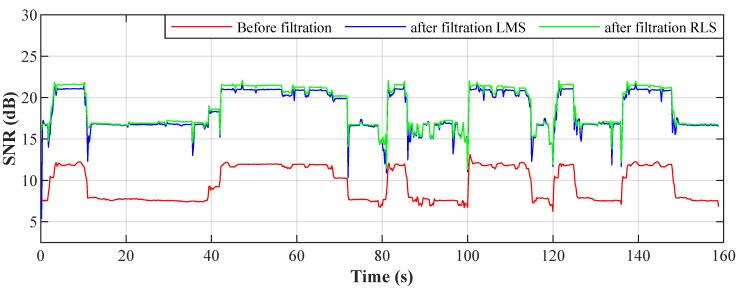
Signal-to-Noise Ratio (SNR) values at intervals of 200 ms.

Figure 25 shows the Signal-to-Noise Ratio (SNR) of the current on the L1 phase before and after filtration. The SNR values were again calculated from time windows: Tw = 200 ms. The decreases and increases in the SNR are, as well as with the THD, caused by switching the LED luminaires on and off. It is clear from the graph that the SNR increased (thus improved) after the filtration, but the behavior of the adaptive algorithms is different. It can be seen that the LMS algorithm achieves a more stable SNR than the RLS algorithm.

#### 4.5.1. Total Harmonic Distortion

THD is defined as the sinusoidal signal distortion and is expressed in percent. It is referred to as the ratio of the sum of the RMS current values of all higher harmonic components to the RMS current value of the fundamental harmonic component.

(15) $$THD_{I}=\frac{\sqrt{I_{2}^{2}+I_{3}^{2}+I_{4}^{2}+\cdots +I_{n}^{2}}}{I_{1}}\cdot 100,$$

where $I_{1}$ is the RMS current value of the fundamental harmonic and $I_{2},\cdots ,I_{n}$ are the RMS current values of higher harmonics.

#### 4.5.2. Signal-to-Noise Ratio

SNR is defined as the useful signal-to-noise ratio and is indicated in decibels. If the SNR is greater than 0 dB, the useful signal is greater than the noise. It can also display the similarity between the signal measured and the ideal (reference) signal.

(16) $$SNR_{\mathrm{OUT}}=10\cdot \log_{10}\left(\frac{\sum_{i=1}^{N-1}{\left[sig_{\mathrm{ideal}}\left(i\right)\right]}^{2}}{\sum_{i=1}^{N-1}{\left[sig_{\mathrm{out}}\left(i\right)-sig_{\mathrm{ideal}}\left(i\right)\right]}^{2}}\right).$$

**Figure 26:**
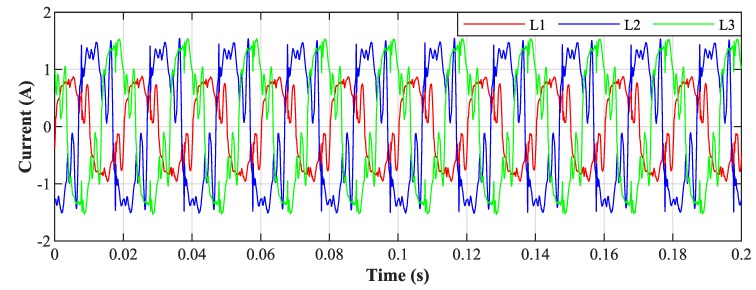
Idle run of test polygon; current waveforms before filtration.

Figure 26 shows the current waveforms in the BROADBAND${}^{\mathrm{LIGHT}}$ test polygon when the LED luminaires are switched off and when the current is still consumed by the power sources switched and the SMART elements, which are permanently powered and consume a nonlinear current. This state is named as *Idle run* of the test polygon. Figure 14 andFigure 15 show the current waveforms after filtration, demonstrating a noticeable improvement in the THD. More accurate THD values before and after filtration are shown in Table 2.

**Figure 27:**
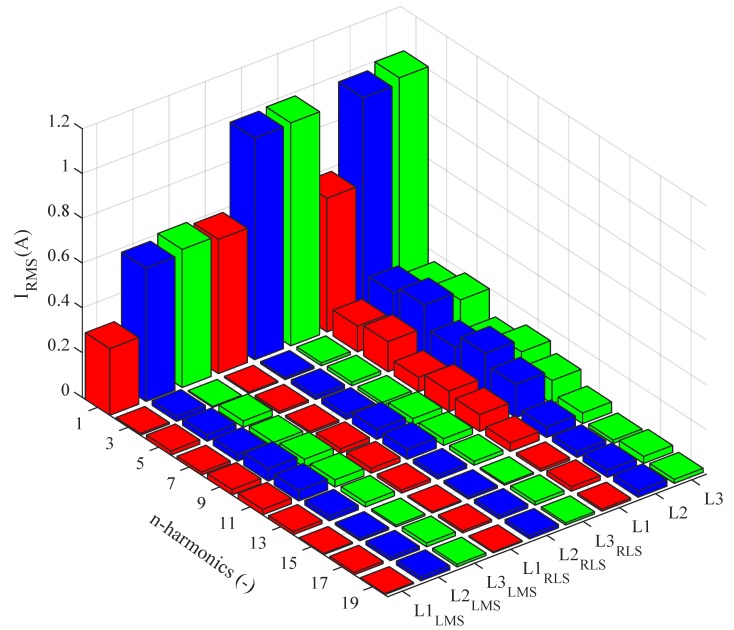
Idle run of test polygon; current spectra before and after application of the adaptive LMS and RLS algorithms.

The current spectra before and after the filter implementation in the *Idle run* state are shown in Figure 27. The graph shows that the LMS has a negative effect on the first harmonic component amplitude, which is caused by the long convergence time of the algorithm. Higher harmonic components are considerably suppressed using both adaptive algorithms.

**Figure 28:**
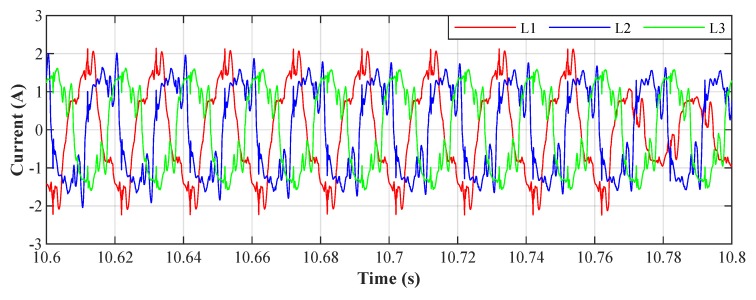
Switched on test polygon; current waveforms before filtration.

Figure 28 shows the current waveforms in the BROADBAND${}^{\mathrm{LIGHT}}$ test polygon when the LED luminaires and the SMART elements are switched on. Figure 16 and Figure 17 show current waveforms after filtration, demonstrating a noticeable improvement in the THD, but the convergence time no longer affects the filtration negatively. More accurate THD values before and after filtration are shown in Table 3.

**Figure 29:**
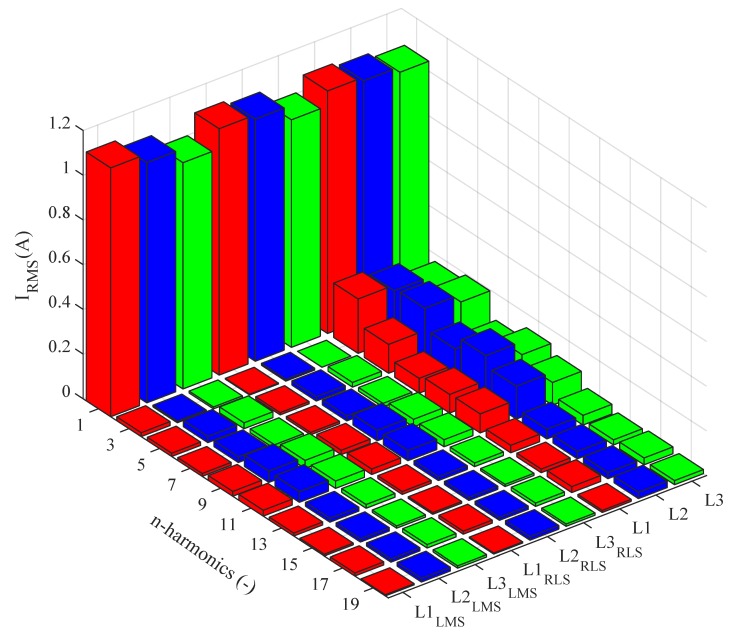
Switched on test polygon; current spectra before and after application of the adaptive LMS and RLS algorithms.

Figure 29 shows the current spectra before and after applying of filtration in switched on state. The graph shows that the LMS has a minimum effect on the first harmonic component amplitude, whereas the RLS has a slightly negative effect on the first harmonic component amplitude. Higher harmonic components were successfully suppressed using both adaptive algorithms.

### 4.6. Implementation on FPGA

One of the important factors influencing the quality of higher harmonic component compensation is the latency between the time of measurement of the relevant values of the load currents and the time when the values measured are processed by the control unit, and subsequently the instantaneous compensation current values are generated by the converter. The latency described should ideally be zero, practically as short as possible (tens, maximum hundreds of microseconds). It is defined as (A) the sum of the time of signal digitization and processing by the control unit and (B) the response time of the output electronics (generating a power compensating current) to the change of the desired output value. The (A) time can be minimized by an efficient and computationally undemanding signal processing algorithm and a high computational performance of the control unit. As part of our research, we implemented an adaptive notch filter with an LMS algorithm, Clarke transformation, and IIR filtration of orthogonal currents into a potential control unit (see Figure 3 and Figure 22). We decided to use virtual instrumentation approach using the NI cRIO platform, which features a FPGA and a microcontroller with a real-time operating system. The cRIO platform was selected specifically because it contains a FPGA that enables implementation of real parallel data processing with high bandpass and the highest level of determinism.

**Figure 30:**
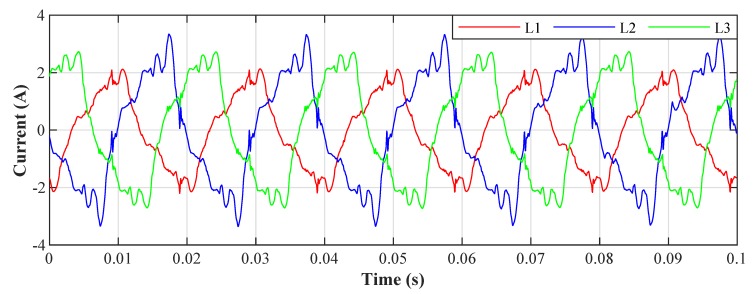
Current waveforms of individual phases before filtration.

**Figure 31:**
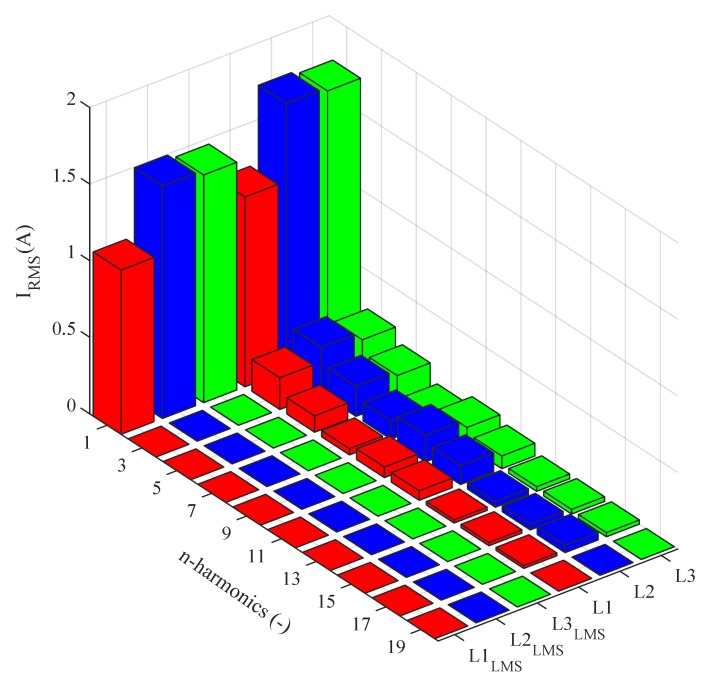
Field Programmable Gate Array (FPGA) Experiment; current spectra before and after application of the adaptive LMS Notch algorithm.

To conduct the experiment we selected the NI myRIO-1900 module intended for the development of control and measurement systems. The system features the Xilinx ZYNQ Z-7010 System on a Chip (SOC), integrating a FPGA and a dual-core ARM processor into a single chip. A variety of peripherals on myRIO-1900 is available to the user: 10 analogue inputs, 6 analogue outputs, and 40 digital input/output lines. A sample of the current waveforms mentioned above (beginning of Section 4) was used as a data source for our experiment. Figure 30 display samples of currents of individual loads, and their frequency characteristics can be seen in Figure 31, marked as *L1*, *L2*, and *L3*.

**Figure 32:**
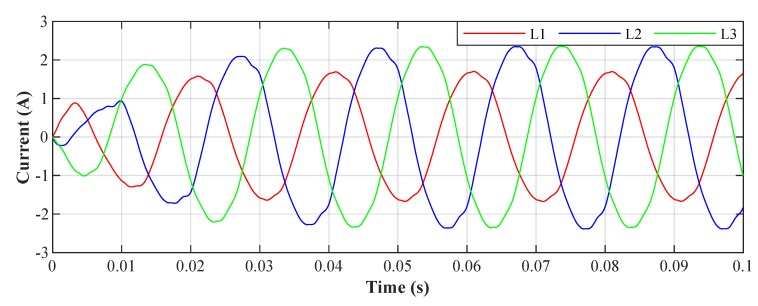
An example of the filtration for all phases for the value μ=0.001, the resulting THDs for the depicted currents correspond to the highlighted column in Table 4. The waveforms of the currents show that the adaptation time is between 40 ms and 50 ms.

The algorithm implemented on the FPGA takes 8 clock cycles of the basic FPGA clock (40 MHz) to process the measured current signals from the moment they are digitized and read from the ADC by the FPGA. Thus, the signal processing time is 8 times the duration of one clock cycle of a 40 MHz clock signal, which corresponds to 8×25 ns =200 ns. This is an extremely short time, representing what we wanted to achieve for this application. NI myRIO includes an ADC with a Successive approximation register (SAR) architecture having a sampling rate of 500 kSa/s. This corresponds to a theoretical conversion time of one sample of 2 μs; when sampling three consecutive channels, the theoretical delay between the signals measured and processed is 6 μs, which is more than sufficient for our project. The implemented system is therefore suitable as a control unit of an active power filter as it is able to control power electronics with a theoretical delay of 6.2 μs. An example of the results obtained from the described experiment for step size μ=0.001 is shown in Figure 31 and Figure 32. Tests were performed on all three phases for five different values of the convergence parameter μ (step size) ranging from 0.00025 to 0.005 with an adaptation time from 5 ms (μ=0.005) to 100 ms (μ=0.00025). A summary of the results is clearly shown in Table 4.

## 5. Discussion

As part of a future work, the team of authors intends to focus on the practical implementation and research of the real conditions of the power network. To create the necessary research conditions, a system for laboratory testing of various operating conditions of the power network was designed and development in cooperation with an industrial partner is planned. The whole system will consist of two parts.

The first part will be a controllable current source for the controlled injection of higher harmonic components into the power supply network, which will serve as a controllable compensator. The second part of the system will be a controllable three-phase load; this part will serve as an imaginary device that generates network interference in the power network. The main advantage of this approach is that further research will not only consist of simulations, but everything will be implemented and verified using real HW.

Another intention of the team is to test different types of adaptive filtration algorithms to obtain a compensating current signal. The effect of the size of the THD as well as the rate of convergence of the algorithm leading to a successful result will be examined.

The key component will be the implementation of control algorithms on the FPGA gate array, which has the main advantage of computational speed, as it enables native parallel data processing. The delay time of the calculation should ideally be up to tens of microseconds so that the quality of compensation of the higher harmonic components was sufficient. A certain disadvantage, which so far prevents mass deployment of the FPGA in active compensators, is the relatively complex implementation of gate array architecture design. As mentioned in the previous paragraphs, the team managed to implement the LMS algorithm in a gate array, which is the simplest one in terms of implementation. The implementation of other algorithms for active filter control is under development.

The above-mentioned system will form a platform for testing SAPF control algorithms. Initially, the testing will be performed under laboratory conditions at the VSB–Technical University of Ostrava campus. Subsequently, implementation in the BROADBAND${}^{\mathrm{LIGHT}}$ test polygon will be performed and operation with real SMART devices will be verified. The system will then be verified also in an industrial environment.

## 6. Conclusions

In this publication, the authors deal with monitoring the quality of electricity, focusing primarily on the issue of SMART street lighting. Particular attention is paid to the testing of individual types of SMART street lighting. Furthermore, there are presented individual algorithms that are used to control active compensators f non-harmonic SAPF waveforms. Subsequently, a unique testing platform for SMART street lighting experiments is presented. As a demonstration of one of the many functionalities, the so-called scenarios are introduced here, where the lighting is controlled via the MQTT communication protocol and the voltage and current data for each phase are collected by means of the measuring system.

In the first part of the practical publication, the authors deal with software implementation of the compensation system using adaptive LMS and RLS algorithms at a simulated converter delay of 100 μs. The function of the adaptive algorithm is fundamentally influenced by its settings, such as the filter length, the step size, and the forgetting factor. Various filter parameter settings were tested by automation and, for ideal adaptive filter settings, a THD improvement of 26.5% for the LMS and 29.4% for the RLS is to be achieved, which is an average THD improvement of 75% for the LMS and 83% for the RLS. The LMS algorithm performs worse and has a longer convergence time than the RLS algorithm, but the RLS algorithm is more demanding computationally and in its implementation.

The second part of the publication deals with the implementation of the concept of active filter control on the FPGA designed, mainly due to the short time needed to calculate the filter algorithm. Due to the implementation on the gate array, a less complicated LMS method was selected. The results show that there was an improvement in all three phases and, therefore, a decrease in the THD value. The best results for the adaptation time were from 40 ms to 50 ms, which corresponds to step size μ=0.001. Here, the THD decreased by 19% on average, which is a relative improvement in the THD by an average of 80%. Lower step size values showed better THD values, but the adaptation time increased up to 100 ms.

## Figures and Tables

**Table 1:** Example of formatting a scenario file.

Index	Time (s)	Method	Power (%)	Wait After (s)
0	0	Full OFF	0	5
1	5	Full ON	100	10
2	15	Full OFF	0	10
3	25	2 - ON	100	3
⋮	⋮	⋮	⋮	⋮
43	153	8,9,10 - OFF	0	3
44	156	11 - OFF	0	3
45	159	Full Off	0	5

**Table 2:** Idle run THD values before and after application of the adaptive LMS and RLS algorithms.

Phase	THD${}_{\mathrm{ORIGINAL}}$(%)	THD${}_{\mathrm{LMS}}$ (%)	THD${}_{\mathrm{RLS}}$ (%)
L1	27.83	4.01	3.62
L2	39.91	8.00	7.02
L3	31.08	6.17	5.25

**Table 3:** THD values in switched on state before and after application of the adaptive LMS and RLS algorithms.

Phase	THD${}_{\mathrm{ORIGINAL}}$(%)	THD${}_{\mathrm{LMS}}$ (%)	THD${}_{RLS}$ (%)
L1	38.06	13.07	6.59
L2	43.33	12.91	7.89
L3	32.37	9.46	5.70

**Table 4:** THD values in the switched on state before and after application of the adaptive LMS algorithm.

	THD (%) without Filtration	THD (%) (*μ* = 0.00025)	THD (%) (*μ* = 0.0005)	THD (%) (*μ* = 0.001)	THD (%) (*μ* = 0.002)	THD (%) (*μ* = 0.005)
L1	22.66	1.25	2.48	4.83	8.86	15.8
L2	27.29	1.6	3.003	5.67	10.27	18.323
L3	21.75	0.9	1.96	3.98	7.49	13.938
Adaptation time (ms)	-	100	60	40	15	5
